# A Modelling Study for Predicting Life of Downhole Tubes Considering Service Environmental Parameters and Stress

**DOI:** 10.3390/ma9090741

**Published:** 2016-09-02

**Authors:** Tianliang Zhao, Zhiyong Liu, Cuiwei Du, Jianpeng Hu, Xiaogang Li

**Affiliations:** 1 Corrosion and Protection Center, University of Science and Technology Beijing, Beijing 100083, China; ustb_tlzhao@163.com (T.Z.); dcw@ustb.edu.cn (C.D.); ustbck0804@163.com (J.H.); lixiaogang99@263.net (X.L.); 2 Key Laboratory for Corrosion and Protection (MOE), Beijing 100083, China; 3 Ningbo Institute of Material Technology & Engineering, Chinese Academy of Sciences, Ningbo 315201, China

**Keywords:** mathematic model, life prediction, downhole tube, environmental parameter, stress

## Abstract

A modelling effort was made to try to predict the life of downhole tubes or casings, synthetically considering the effect of service influencing factors on corrosion rate. Based on the discussed corrosion mechanism and corrosion processes of downhole tubes, a mathematic model was established. For downhole tubes, the influencing factors are environmental parameters and stress, which vary with service duration. Stress and the environmental parameters including water content, partial pressure of H_2_S and CO_2_, pH value, total pressure and temperature, were considered to be time-dependent. Based on the model, life-span of an L80 downhole tube in oilfield Halfaya, an oilfield in Iraq, was predicted. The results show that life-span of the L80 downhole tube in Halfaya is 247 months (approximately 20 years) under initial stress of 0.1 yield strength and 641 months (approximately 53 years) under no initial stress, which indicates that an initial stress of 0.1 yield strength will reduce the life-span by more than half.

## 1. Introduction

Failure of the downhole tube and casing is a problem constantly disturbing oil production. Once the tube or casing, especially the tube, is corroded to perforation or cracking, the oil well may face production suspension or may even be discarded. Since the downhole environment is severely corrosive and complex, it is hard to protect tubes and casings from aggressive corrosion. However, we will have more choices if we can foreknow when they fail. With knowing service life of tubes and casings, engineers can use different strategies regarding material selection, operation optimization and periodic maintenance [1,2]. Thus, their values are made full use of during their service time so that unnecessary cost can be avoided. Accidental risk can also be controlled effectively [3]. Therefore, it is really of great significance to carry out an investigation on the service life prediction of downhole tubes and casings.

Over the past decades, much effort has been devoted to prediction, and researchers have proposed many models for prediction. The way of establishing model is various and can be divided into data mining models, failure-mechanism-based models, and combination of the two [4,5,6,7,8,9,10,11]. The data mining models do not care about the mechanism and processes of corrosion. They are mainly based on statistics from vast amounts of field data; for example, Hu et al. [4] proposed a cross-scale life-time prediction model for oil tubes mainly based on statistical theory and used Monte-Carlo method as the numerical simulation method. Caleyo et al. [5] also used Monte-Carlo method to predict the time evolution of pit depth on underground pipelines. Yang and Wang [6] proposed a model based on the grey system theory to predict the residual life of submarine tubes. These models usually give relatively accurate prediction. However, they are of poor operability as they rely on large amounts of field data and experience. The models based on failure mechanism overcome this shortcoming. They require a scientific understanding of corrosion mechanism and processes. For example, Zhang [7] developed a residual life prediction model based on elastic-plastic fracture mechanics. He took the combined effects of internal pressure and axial force into consideration as the main influences, but ignored their variation with service time in his model. Then, Parkins [8] further applied stress corrosion crack growth kinetics to the prediction. For stainless steel, Song [3] developed a mathematical model based on the film rupture and repassivation mechanism. However, the above studies mainly focused on the individual process of crack propagation, which accounts for only a small part of the whole service life. Although these models seem theoretically reasonable, the results are always away from practice. Models combining data mining and failure mechanism, which overcome their respective disadvantages, have also been proposed. Krouse et al. and Laycock et al. [9,10,11] established a model characterizing the relationship between maximum deepness of local corrosion and time with method of extreme value statistic. Melchers [9] also attempted to model variation of pit depth with elapsed exposure time and influencing factors. His model was based on reasonable assumptions from corrosion science and field observation and gave results through statistics of maximum pit depth. Although his work is widely recognized, his model ignored the effect of influencing factors on pit depth.

Corrosion rate, based on corrosion mechanism and usually being applied as corrosion losses or rate of pit growth, is one main type of data used in modelling [12]. It is not easy to establish a model combining corrosion data and corrosion mechanism, especially when the effect of time and other influencing factors are taken into consideration. Development of such model requires a combination of scientific understanding of corrosion processes and sound approaches to mathematical modelling [13]. For easier use in practice, models are usually over-simplified using a time-independent corrosion rate, such as initial corrosion rate or average corrosion rate, or ignoring the effect of environmental and stress evolution [14]. Actually, corrosion rate mostly act as a non-linear functions of time and other influence factors [15]. Models of irrational simplicity usually result in serious low predicted life-span or error judgment about SCC critical conditions. The former will lead to uneconomic material-selection and the later will lead to serious accidents [16].

For downhole casings and tubes, water content, partial pressure of H_2_S and CO_2_, pH value, total pressure, temperature, and the axial stress induced by self-weight are the main influencing factors of corrosion rate. The present work proposes a new prediction model or method synthetically considering those factors mentioned above. This model or method is closer to practice and avoid the above-mentioned problems. Two criteria for failure judgment, the thickness criterion and the strength criterion, aiming at the failure caused by wall thickness thinning and strength loss, respectively, have also been proposed.

## 2. Criteria for Failure Judgment

It is generally known that there are mainly two types of integrity loss of downhole tubes [17]. One type is perforation and leakage induced by local corrosion of tube wall. In this form, lives of the tubes are limited by thickness of local corrosion position, namely tubes are assumed to fail when the wall is corroded to a certain thickness. The well-known ASME B31G Standard assumes that tubes fail when deepness of the defect is greater than 80% of the wall thickness [18]. Thus, failure of downhole tubes can be judged with the following equation.

(1) $$L_{0}-\Delta L\geq nL_{0}$$

Equation (1) can be called the thickness criterion (TC). *L*_0_ is the initial thickness of tube wall. ∆*L* is the corroded thickness during service and can be expressed as the functions of service time *t*. *n* is the coefficient for thickness safety, the value of which is 0.2 according to ASME B31G Standard [18].

The other type is cracking induced by stress concentration. In this form, the tubes are assumed to fail when the suffered stress exceeds the allowable maximum stress (*S*_c_). According to the residual strength criterion [19], failure of downhole tubes can be judged with the following equation.

(2) $$mS_{0}\frac{L_{0}}{L_{0}-\Delta L}\leq S_{c}$$

where *m* is the coefficient for strength safety, the value of which can be set as 1.5 empirically. *S*_0_ is the initial stress posed on tube wall. It is determined by the initial service status. Equation (2) can be called the strength criterion (SC). It is noted that the SC is suitable both for general corrosion and local corrosion. The corrosion form will significantly affect the value of *S*_c_. This will be discussed in detail in Section 6.1.

## 3. Expression of Life Prediction Model

Naturally, the tube wall reduces as service time goes on. Thus, ∆*L* is the function of service time, *t*.

(3) $$\Delta L=\int_{0}^{t}Cdt^{'}$$

where *C* is the corrosion rate of oil tube and a function of service time *t*. Its influence factors includes the suffered stress (*S*), water content (the proportion of water in oil–water mixture, *W*), temperature (*T*), the partial pressure of H_2_S ($P_{H_{2}S}$), the partial pressure of CO_2_ ($P_{{\text{CO}}_{2}}$), pH value and the concentration of Cl^−^ ($C_{{\text{Cl}}^{-}}$). Therefore, *C* can be expressed as below.

(4) $$C=F\left(S,W,T,P_{H_{2}S},P_{{\text{CO}}_{2}},\text{pH},C_{{\text{Cl}}^{-}},\cdots \right)$$

where *S*, *W*, *T*, *P*_H2S_, *P*_CO__₂_, pH, *C*_Cl−_ etc. are also functions of *t*. Their dependences on *t* can be obtained through monitoring on service environment over time. They are expressed as follows:

(5) $$S=f_{1}\left(t\right)$$

(6) $$W=f_{2}\left(t\right)$$

(7) $$T=f_{3}\left(t\right)$$

(8) $$P_{H_{2}S}=f_{4}\left(t\right)$$

(9) $$P_{{\text{CO}}_{2}}=f_{5}\left(t\right)$$

Therefore, Equation (4) is transformed to the below equation.

(10) C=G(t)

Plugging Equation (10) into Equation (3), dependence of ∆*L* on *t* can be obtained as follows.

(11) $$\Delta L=\int_{0}^{t}G\left(t^{'}\right)dt^{'}=H\left(t\right)$$

Combining Equations (1), (2) and (11), service life of oil tube can be obtained. The life prediction model is mainly expressed by Equation (11). It provides a new way of life-span prediction of downhole tubes, namely, synthetically considering the evolution of service environment and stress levels. Thus, it is more in accordance with the engineering practice. Simultaneously, this also makes it have some disadvantages inevitably. The specific expression of Equation (4) becomes difficult to work out when it is a multivariate function. However, we can still approach it through the method of multivariate function interpolation described in literature [20].

## 4. Experimental

### 4.1. Material and Medium

**Figure 1:**
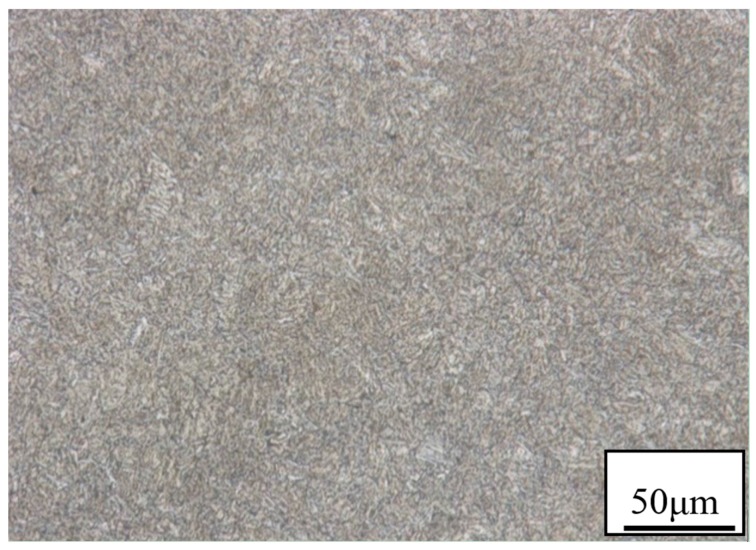
Microstructure of L80 steel.

Specimens used in this work were made of L80 tubing steel with chemical composition (wt %): 0.32 C, 0.19 Si, 1.35 Mn, 0.24 Cr, 0.033 Cu, <0.10 Mo, 0.015 S, 0.0088 P and Fe balance. Its microstructure is shown in Figure 1. It reveals that L80 steel is mainly made of fine bainite. That makes L80 steel have a pretty good mechanical performance: yield strength (*σ*_s_) of 675 MPa, ultimate tensile strength (*UTS*) of 797 MPa, elongation (*δ*_0_) of 22.3% and reduction-in-area (*RA*) of 69.5%.

**Figure 2:**
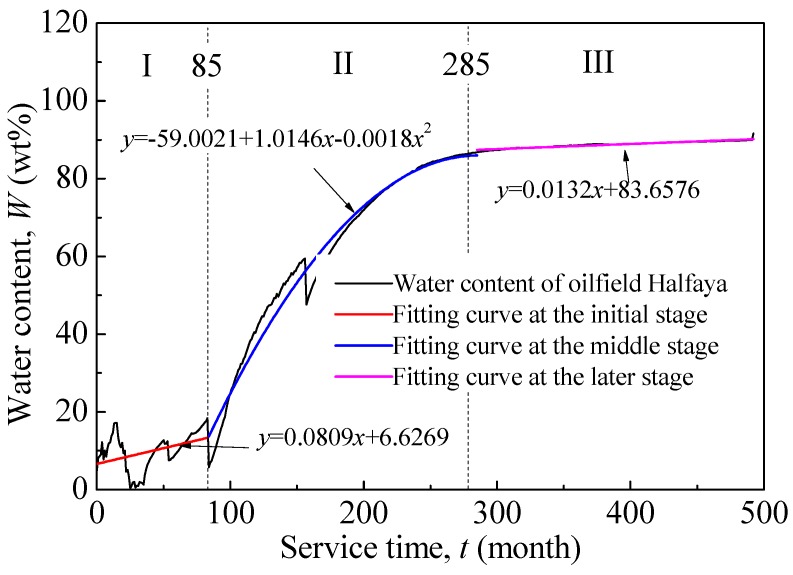
Dependence of water content of oilfield Halfaya on service time.

The mediums were oil–water mixtures prepared in different proportions of oil and mineralized water to simulate the water content of oilfield Halfaya at different service time. Halfaya is a typical oilfields in Middle East. Dependence of its water content on service time is shown in Figure 2. Accordingly, the proportions were set at 5 wt %, 30 wt %, 50 wt %, 80 wt %, and 100 wt %. Linear fittings corresponding to different service stages were also given. The mineralized water was prepared according to chemical composition shown in Table 1. The oil is crude oil from oilfield Halfaya. The oil and the mineralized water were mixed, and stirred for 12 h to form an oil-in-water or water-in-oil emulsion.

Practical monitoring result of oilfield Halfaya shows that partial pressure of H_2_S and CO_2_, pH value, total pressure, and temperature downhole will be steady soon after the oilfield is put into production. Although pH value is generally supposed to have a great effect on corrosion rate, it is not concluded in the influencing factors because acidification of mineralized water is neutralized by constantly injected water. Thus, water content and stress is assumed to be only two variables during the whole service period. The partial pressure of H_2_S and CO_2_, total pressure and temperature are approximately 0.15 MPa, 1.1 MPa, 10 MPa and 80 °C, respectively. The experimental conditions were set in accordance to these results.

### 4.2. Potentiodynamic Polarization Measurement

Considering that water content and stress would have an impact on the corrosion behaviour of L80 steel, potentiodynamic polarization measurement was conducted on the specimens under different water contents and stresses. A thermostatic autoclave with conventional three-electrode system was used to perform the measurements under different water contents. The specimens were cut into plates with sized of 10 mm × 10 mm × 3 mm and sealed with epoxy resin, leaving a working square of 10 mm × 10 mm exposed. The prepared medium was added into the autoclave and experimental conditions were applied with reference to Section 4.1.

The measurements under different stresses were performed with a CORTEST slow strain rate test system (CORTEST, Willoughby, OH, USA), which also has a three-electrode system. The specimens were commonly-seen tensile plates. They were sealed with high-temperature silicone, leaving a square of 5 mm × 10 mm in the working segment exposed. Prior to the measurements, the specimens were preloaded to different stress levels, i.e., 0.5 *σ*_s_, 0.8 *σ*_s_ and 1.0 *σ*_s_ (here it is assumed that *σ*_s_ is equal to the proof stress). The medium of 80 wt % water content was then added into the autoclave and experimental conditions were applied with reference to Section 4.1. The polarization measurements would not start at these particular stress levels until corrosion potential of the specimen was stable.

All tests were carried out with a potential scanning rate of 1 mV/s. The medium was de-oxygenated by purging nitrogen gas for 2 h before test. The L80 steel specimen was used as working electrode, SCE as reference electrode and a platinum plate as counter electrode. The entire specimen was ground sequentially to 1000 grit emery paper.

### 4.3. Immersion Test

**Figure 3:**
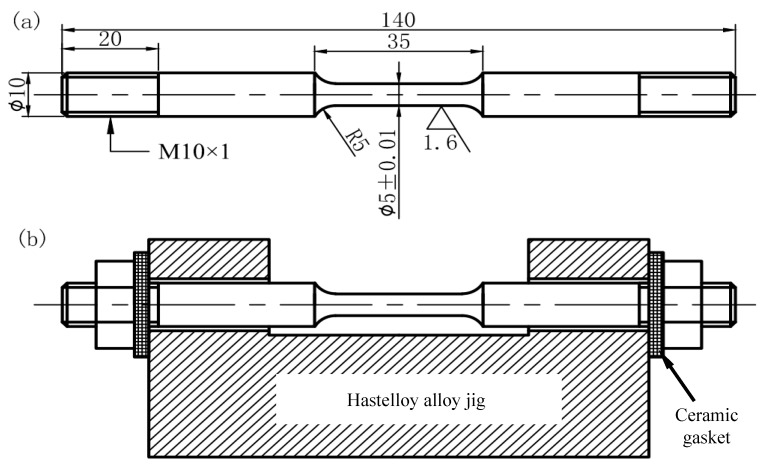
Sizes of (**a**) the specimen and (**b**) the assembling jig used for preloading.

Immersion test was performed on specimens with different stress levels. Before immersion, specimens were cleaned and weighed. They were then preloaded to different stress levels with a WDML-30KN (LETRY, Xi’an, China) electron-tensile tester and fixed with assembling jigs and nuts. The levels were 0 *σ*_s_, 0.5 *σ*_s_, 0.8 *σ*_s_ and 1.0 *σ*_s_. Size of the specimen and the jig are shown in Figure 3. The jig was made from Hastelloy alloy, which has excellent rigidity and corrosion resistance. Ceramic gaskets were used to ensure the specimen insulated from the jig. Both ends of the specimen, the nuts and the arc transition sections were coated with temperature-resistant silica gel, leaving the necking sections exposed. The prepared medium and specimens were then added into a thermostatic autoclave and the medium was de-oxygenated by purging nitrogen gas for 2 h. The experimental conditions were applied with reference to Section 4.1. The immersion time lasted for 720 h.

After immersion, the specimens were cleaned with acetone and distilled water and then weighed. Corrosion rates corresponding to different preloading stresses were calculated according to the weight loss. Corrosion morphologies of the necking sections were observed with VHX 2000 (Keyence, Osaka, Japan) stereomicroscope. Then tensile tests were performed on those corroded specimens.

### 4.4. Tensile Tests after Immersion

Tensile tests were conducted on other parallel immersed specimens according to ASTM E8M-09 (ASTM, West Conshohocken, PA, USA) [21]. The tensile rate was 0.007/min and 0.05/min before and after the specimen yielded, respectively. Then, the stress–strain curves, elongations and reductions in area were obtained.

## 5. Results

### 5.1. Potentiodynamic Polarization Curves

**Figure 4:**
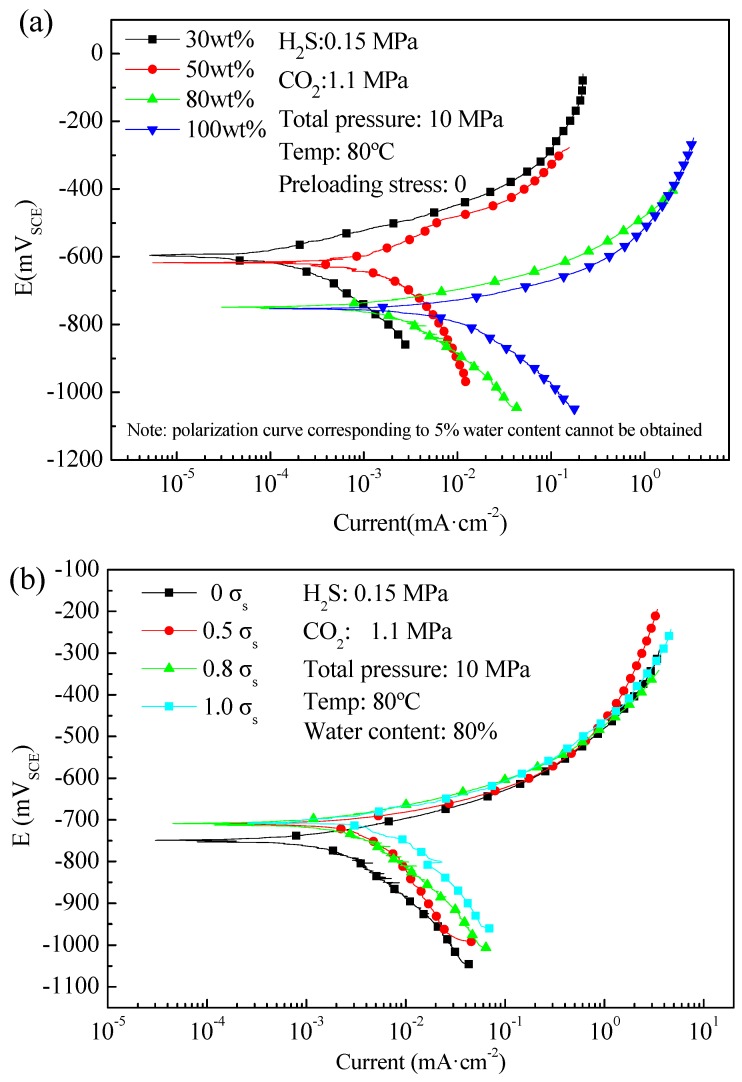
Potentiodynamic polarization curves of L80 steel measured under different: (**a**) water contents; and (**b**) preloading stresses.

Figure 4 shows the potentiodynamic polarization curves of L80 steel measured under different water contents and preloading stresses. It is seen that both water content and preloading stress affect significantly the electrochemical polarization behaviour of L80 steel. However, shape of the potentiodynamic polarization curves does not change with the increasing of water content or preloading stress. Generally, the smooth shape indicates that anodic and cathodic processes are controlled by electrochemical reaction step. Increasing the water content or preloading stress does not affect the corrosion type of L80 in test medium except for the corrosion potential and corrosion current density. It was also found in previous studies [22,23,24] that the anodic and cathodic processes of carbon steel in similar environment (the high pressure H_2_S/CO_2_ environment) are activation controlled. It is also noted that cathodic reduction reactions (hydrogen reduction) are promoted by both increasing of water content and preloading stress (see the cathodic polarization segment in Figure 4a,b). Increasing of water content increases the exposed area of steel to mineralized water, thereby promoting the cathodic reactions. The cathodic reactions increase with increasing of preloading stress, which can be explained by local additional potential model (LAPM) [25]. In elastic stress region, local stress concentration may occur at micro-defects, such as twins, micro-cracks, inclusions, etc. Dislocation slip could thus occur at these sites. Dislocation emergence points and slip steps introduce active sites on the steel surface, and electrons would flow and concentrate at these sites to result in a local charging effect. As a result, a local additional potential (LAP, generally positive) is generated when the steel surface is exposed in a solution. With an increasing of stress, the LAP increases, resulting in the increasing of cathodic reaction current. Therefore, with the above two factors increasing, risk of hydrogen brittlement or hydrogen induced cracking may be increased. Mechanical properties of L80 after immersed in the test medium need to be investigated.

### 5.2. Corrosion Morphology

**Figure 5:**
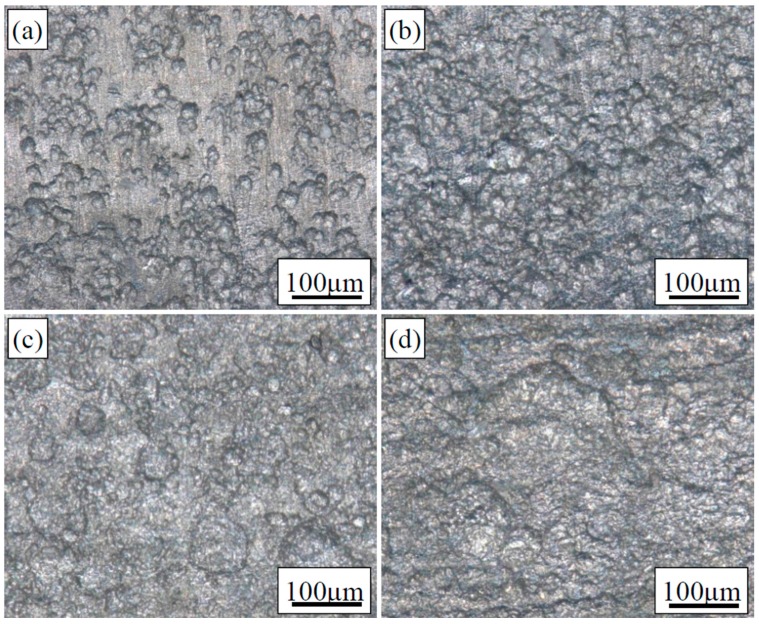
Corrosion morphology of L80 steel after immersed in the medium of 80 wt % water content for under preloading stresses of: (**a**) 0 *σ*_s_; (**b**) 0.5 *σ*_s_; (**c**) 0.8 *σ*_s_; and (**d**) 1.0 *σ*_s_.

Figure 5 shows corrosion morphologies of L80 steel after immersed in the medium of 80 wt % water content for 720 h under preloading stresses of: Figure 5a 0 *σ*_s_; Figure 5b 0.5 *σ*_s_; Figure 5c 0.8 *σ*_s_; and Figure 5d 1.0 *σ*_s_. It can be seen that many shallow pits are uniformly distributed on the surface and no crack nucleates. Those shallow pits are not deepened, but gradually connected to each other along with the increasing of preloading stress. The connected pits transform into a relatively uniform corrosion. The corrosion morphology partially confirms the results of potentiodynamic polarization measurement, i.e., L80 steel will corrode uniformly in downhole environment, even with stress of 1.0 *σ*_s_.

### 5.3. Tensile Properties after Immersion

**Figure 6:**
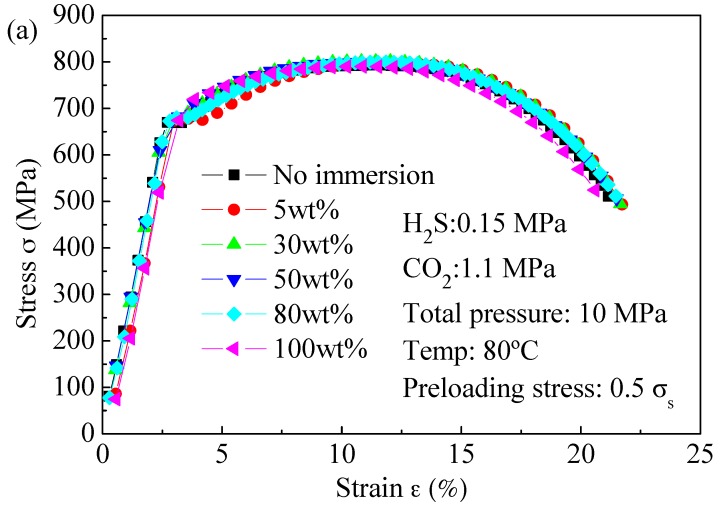

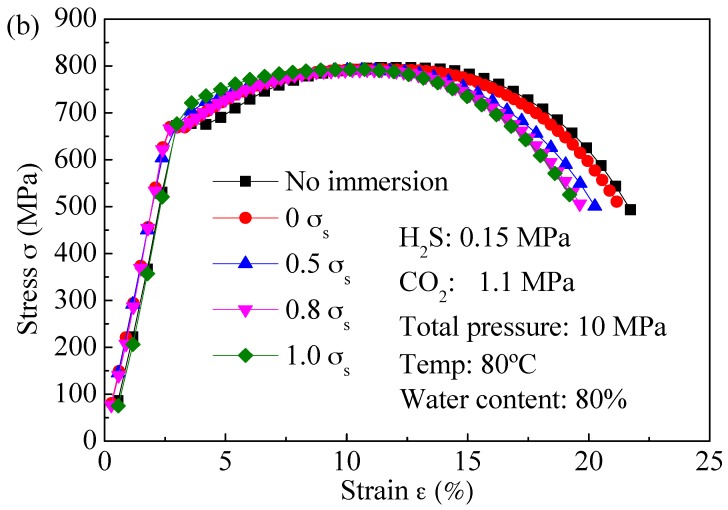
Dependence of stress–strain curve of L80 steel on: (**a**) water content; and (**b**) preloading stress after immersion in the test medium for 720 h.

Figure 6 shows the stress–strain curves of L80 steel tested after immersion in the test medium for 720 h under different water contents and preloading stresses. It is seen from them that both water content and preloading stress have little influence on the mechanical properties of L80 steel, except slightly decreasing the elongation. The yield strengths in all conditions remain the same as that in air. It can be assumed that *S*_c_ is equal to the yield strength in air.

**Figure 7:**
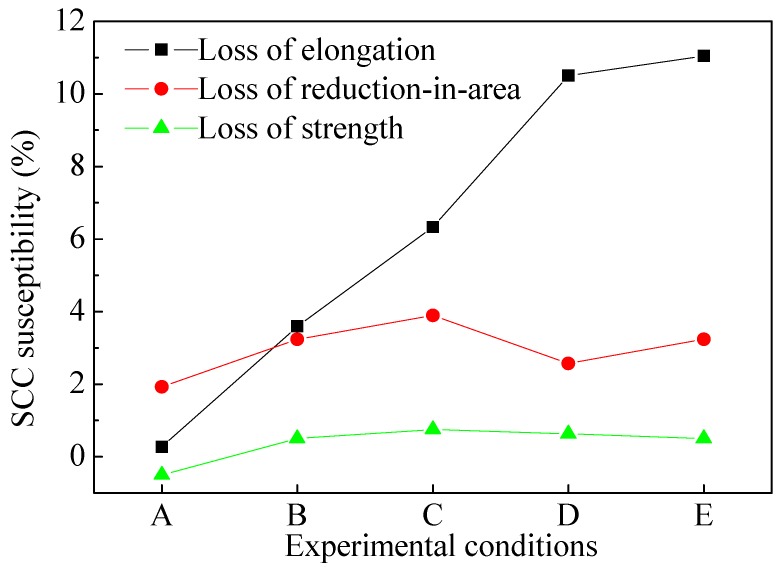
Variation of SCC susceptibility of L80 steel after immersion for 720 h with water content and preloading stress (A: 5 wt %, 0 *σ*_s_; B: 80 wt %, 0 *σ*_s_; C: 80 wt %, 0.5 *σ*_s_; D: 80 wt %, 0.8 *σ*_s_; and E: 80 wt %, 1.0 *σ*_s_).

Figure 7 shows the SCC susceptibility of L80 steel varying with water content and preloading stress. It can be seen that loss of elongation, loss of reduction-in-area and loss of strength are all less than 12%. It indicates that L80 steel have little tendency towards stress corrosion in downhole environment.

### 5.4. Corrosion Rates

**Figure 8:**
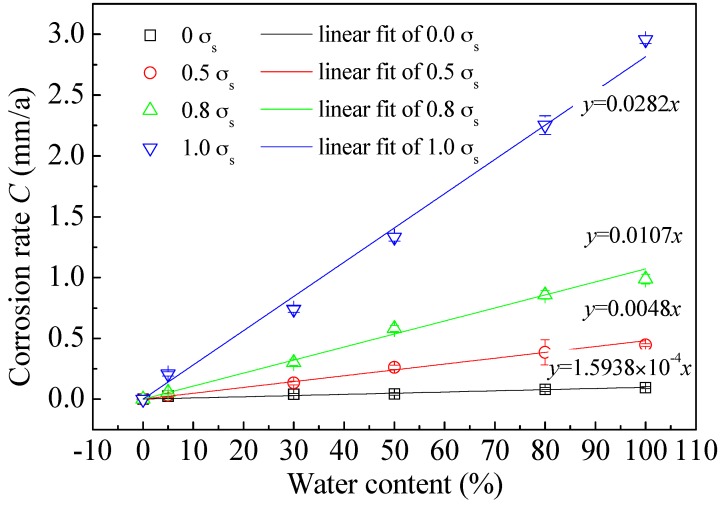
Corrosion rates of L80 steel and their fittings as a function of water content under different preloading stress.

Table 2 lists the corrosion rates of L80 steel under different water contents and different preloading stresses. Those data are replotted in Figure 8. It is seen that corrosion rate (in the form of average thinning rate) of L80 steel increases linearly with increasing water content when the preloading stress is constant. Relationship expressions between corrosion rate and water content under different preloading stresses are labelled on the corresponding fitting lines.

**Figure 9:**
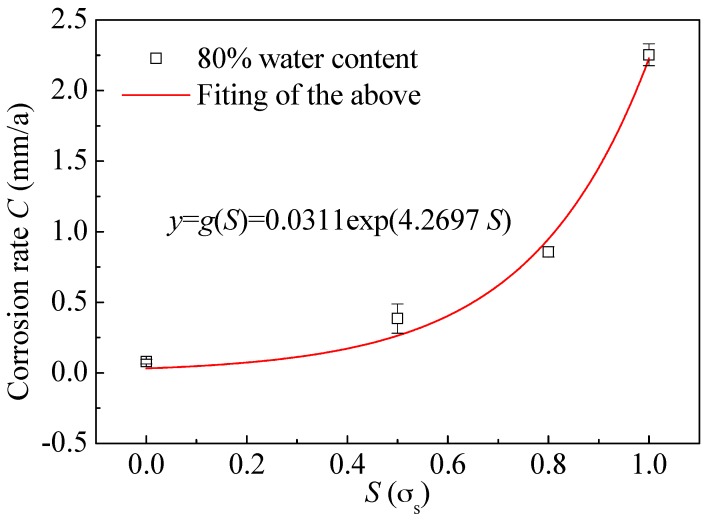
Dependence of corrosion rate of L80 steel at 80% water content on preloading stress.

Figure 9 shows the dependence of corrosion rate at 80% water content on preloading stress. Data are fitted with exponential function. The expression is as follows:

(12) y=g(S)=aexp(b⋅S)

where fitting values of *a* and *b* are 0.0311 and 4.2697, respectively. Relationship between corrosion rates at other water content and preloading stress can be also expressed by Equation (12).

## 6. Life Prediction of L80 Oil Tube in Halfaya

### 6.1. Suitability of the Model

Potentialdynamic polarization curves (Figure 4) and corrosion morphologies (Figure 5) in last section reveal that L80 steel is corroded uniformly in simulated medium of Halfaya downhole environment. Generally, uniform corrosion means that the oil tube will not suffer great stress concentration. It is reasonable to assume that the true stress is equal to the nominal stress (*S*_n_). *S*_n_ is defined as the load (*F*) divided by the actual cross-sectional area (*A*). For oil tube, *F* is generally equal to gravity of itself and *A* is time-dependent. When the true stress reaches the proof stress, which is regarded as the threshold of local instability and usually equivalent to the yield strength, *S*_n_ also reaches the allowable maximum stress (*S*_c_ in Equation (2)). Here, *S*_c_ is equal to the proof stress, i.e., the yield strength.

However, uniform corrosion is not the only corrosion form for the tubes. Local corrosion and stress corrosion also widely exist on the tubes. For local corrosion, such as pitting, it will induce a stress concentration around itself. The local stress will be obviously greater than *S*_n_. Here, *S*_c_ is no longer equal to the proof stress. Because the local stress will reach the proof stress before *S*_n_ does. *S*_c_ should be equal to the nominal stress at the point when the local stress reaches the proof stress. It can be obtained by methods of finite element modelling or introducing an appropriate coefficient. If stress corrosion induces the steel initiating crack before the local stress reaches the proof stress, *S*_c_ should be equal to the nominal stress at the point when the steel initiates crack.

In brief, the model can be suitable not only for uniform corrosion (or general corrosion) but also local corrosion and stress corrosion by adjusting value of *S*_c_ according to the actual corrosion form and mechanism.

### 6.2. Mathematical Process

**Figure 10:**
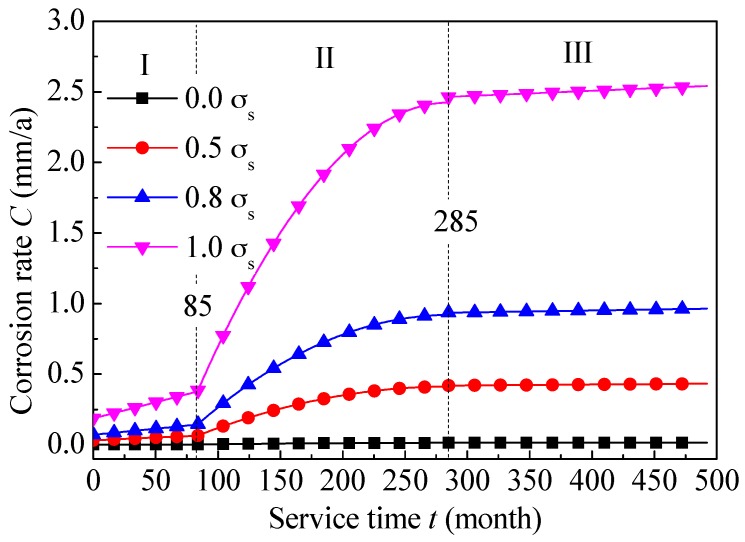
Corrosion rates of L80 steel as a function of service time under different preloading stress.

Figure 10 shows the variation of corrosion rate with service time under different preloading stress. It is obtained by iterating ordinate function of Figure 2 into abscissa variable of Figure 10. Expressions of 0.0 *σ*_s_ and 1.0 *σ*_s_ corresponding to the three stages are given as follows (Equations (13)–(18)).

(13) $$C_{I}=1.2886\times 10^{-5}t+1.0562\times 10^{-3},t\leq 85$$

(14) $$C_{\text{II}}=-2.8369\times 10^{-7}t^{2}+1.6170\times 10^{-4}t-9.4035\times 10^{-3},85<t\leq 285$$

(15) $$C_{\text{III}}=2.0996\times 10^{-6}t+0.0133,t>285$$

(16) $$C_{I}^{'}=2.2800\times 10^{-3}t+0.1869,t\leq 85$$

(17) $$C_{\text{II}}^{'}=-5.0196\times 10^{-5}t^{2}+0.0286t-1.6639,85<t\leq 285$$

(18) $$C_{\text{III}}^{'}=3.7139\times 10^{-4}t+2.3591,t>285$$

As previously mentioned, temperature and partial pressure of H_2_S and CO_2_ are steady during service time. Therefore, *T*, *P*_H2S_ and *P*_CO__₂_ in Equation (4) can be seen as constants and *C* is mainly affected by *S* and *W*. In order to make the process feasible, Equation (4) is simplified as follows:

(19) C=F(S,W)=f(S)⋅g(W)

**Figure 11:**
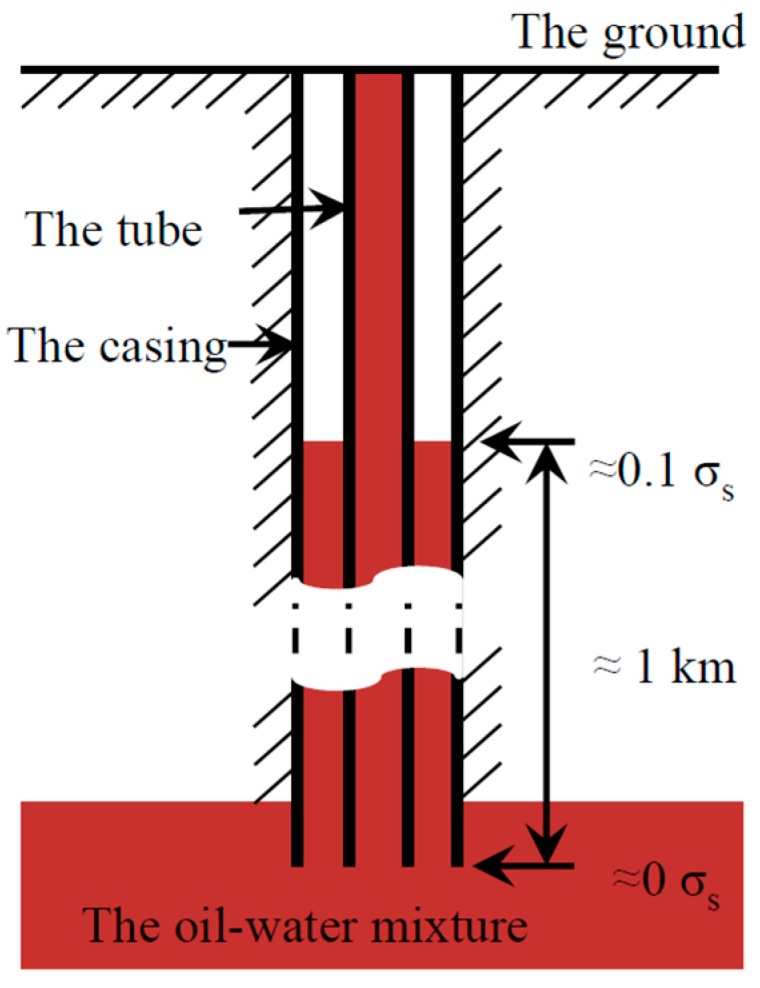
The initial stresses that different parts of the downhole tube or casing suffer.

The initial stress will have a great influence on corrosion rate. Life-span of tubes will be far different when the initial stress is zero or not. The initial stresses that different parts of the downhole tube or casing suffer are illustrated in Figure 11. Generally, level of the oil–water mixture can reach one-kilometre high from the bottom of the tube. Stress at level of the oil–water mixture is approximately equal to 0.1 *σ*_s_, which is roughly calculated from weight of one-kilometre-long tube. The stress at the bottom is zero. Wall thickness and diameter of the downhole tube are 9 mm and 200 mm, respectively.

#### 6.2.1. When the Initial Stress is Zero

If the initial stress is zero, the stress that oil tube suffers in whole service time is also zero. Relationship between *C* and *t* should be subject to Equations (13)–(15). Therefore, ∆*L* can be obtained by substituting Equations (13)–(15) into Equation (3).

(20) $$\Delta L=\{\begin{array}{c} \int_{0}^{t}C_{I}dt^{'},t^{'}>85\\ \int_{0}^{85}C_{I}dt^{'}+\int_{85}^{t}C_{\text{II}}dt^{'},85<t^{'}\leq 285\\ \int_{0}^{85}C_{I}dt^{'}+\int_{85}^{285}C_{\text{II}}dt^{'}+\int_{285}^{t}C_{\text{III}}dt^{'},t^{'}>285 \end{array}$$

#### 6.2.2. When the Initial Stress is 0.1 *σ*_s_

It is already known that *C* is the function of *W* and *S*, and *W* and *S* are the functions of *t*. Thus, *C* can be expressed as follows.

(21) $$C=f\left(S\right)\cdot g\left(W\right)=f_{1}\left(t\right)\cdot f_{2}\left(t\right)$$

Thus, if only expressions of *f*_1_ and *f*_2_ are obtained, the *C* as a function of *t* is achieved.

##### The Initial Stage (0–85th Months)

In the initial stage, there is a linear relationship between *C* and *t* when the preloading stress is constant. Thus, *f*_2_ in the initial stage can be assumed as:

(22) $$f_{2}=At+B$$

where A and B are constants. Since the axial force that the oil tube suffers is equal to the gravity, there is:

(23) $$\left(S+dS\right)\left(L_{0}-Cdt\right)=S_{0}L_{0}$$

Equation (23) can be transformed into:

(24) $$L_{0}dS=S_{0}Cdt=S_{0}f\left(S\right)f_{2}\left(t\right)dt$$

(25) $$\frac{dS}{f\left(S\right)}=\frac{S_{0}}{L_{0}}f_{2}\left(t\right)dt$$

Equations (12) and (22) are substituted into Equation (25), and then there is:

(26) $$\frac{dS}{a\exp\left(bS\right)}=\frac{S_{0}}{L_{0}}\left(At+B\right)dt$$

Both sides of Equation (26) are infinitely integrated. Then there is:

(27) $$\exp\left(bS\right)=-\frac{L_{0}}{abS_{0}\left(\frac{A}{2}t^{2}+Bt+C_{1}\right)}$$

where C_1_ is a constant. Equation (27) is substituted into Equation (12), and then there is:

(28) $$f\left(S\right)=-\frac{L_{0}}{bS_{0}\left(\frac{A}{2}t^{2}+Bt+C_{1}\right)}$$

Then, Equations (28) and (22) are substituted into Equation (21).

(29) $${C_{I}}^{\prime }=-\frac{L_{0}}{bS_{0}}\cdot \frac{At+B}{\frac{A}{2}t^{2}+Bt+C_{1}}$$

Thus, ∆*L* of the initial stage (∆*L*_1_) can be obtained according to Equation (3).

(30) $$\Delta L_{1}=\int -\frac{L_{0}}{bS_{0}}\cdot \frac{At+B}{\frac{A}{2}t^{2}+Bt+C_{1}}dt=-\frac{L_{0}}{bS_{0}}\ln\left|\frac{A}{2}t^{2}+Bt+C_{1}\right|+D$$

where D is a constant. When value of *S*_0_ is 1.0 (*σ*_s_), the equation below can be set up through combining Equations (12), (16), (21) and (22).

(31) $$C=\left(At+B\right)\cdot a\exp\left(b\right)=2.28\times 10^{-3}t+0.1869$$

By substituting value of *a* and *b* into Equation (31), the value of A and B are obtained: A = 1.0253 × 10^−3^, and B = 0.0840. When value of *t* is 0, value of *S* is 0.1. Thus, the equation below can also be obtained through combining Equations (12), (21), (22) and (29).

(32) $$C=B\cdot a\exp\left(0.1b\right)=-\frac{10L_{0}B}{bC_{1}}$$

where the value of *L*_0_ is 9 (mm) in this work. Therefore, value of C_1_ can be obtained: C_1_ = −442.2359. When value of t is zero, value of ∆*L*_1_ should be zero too. Therefore, D can be worked out according to Equation (30): D = 128.4085.

##### The Middle Stage (85th–285th Months)

In the middle stage, there is a quadratic relationship between *C* and *t* when the preloading stress is constant. Thus, *f*_2_ in the middle stage can be assumed as:

(33) $$f_{2}={\text{A}}^{\prime }t^{2}+{\text{B}}^{\prime }t+{\text{C}}_{1}^{\prime }$$

As same as the process in the initial stage does, expression of ∆*L* of the middle stage (∆*L*_2_) can be derived:

(34) $$\Delta L_{2}=-\frac{L_{0}}{bS_{0}}\ln\left|\frac{{\text{A}}^{\prime }}{3}t^{3}+\frac{{\text{B}}^{\prime }}{2}t^{2}+{\text{C}}_{1}^{\prime }t+{\text{D}}^{\prime }\right|+{\text{E}}^{\prime }$$

where values of A*ʹ*, B*ʹ*, C_1_*ʹ*, D*ʹ* and E*ʹ* can be worked out in the way similar to the initial stage: A*ʹ* = −2.2574 × 10^−5^, B*ʹ* = 1.2862 × 10^−2^, C_1_*ʹ* = −0.7483, D*ʹ* = −442.2359 and E*ʹ* = 129.9444.

#### 6.2.3. The Last Stage (285th Month Onward)

As same as the process in the initial stage does, expression of ∆*L* of the last stage (∆*L*_3_) can be derived:

(35) $$\Delta L_{3}=-\frac{L_{0}}{bS_{0}}\ln\left|\frac{{\text{A}}^{\prime \prime }}{2}t^{2}+{\text{B}}^{\prime \prime }t+{C_{1}}^{\prime \prime }\right|+{\text{D}}^{\prime \prime }$$

where values of A*ʺ*, B*ʺ*, C_1_*ʺ* and D*ʺ* are 3.7139 × 10^−4^, 1.0609, −442.2359, and 115.2410, respectively.

### 6.3. Results of Life Prediction

**Figure 12:**
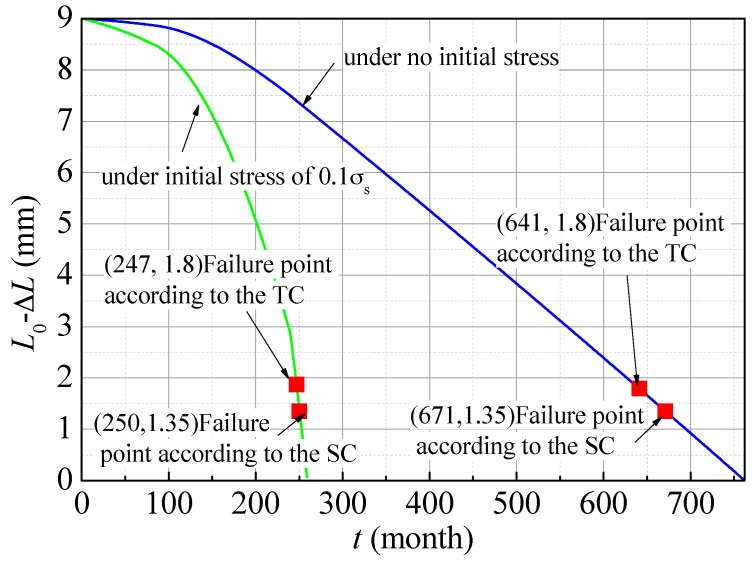
Variation of residual wall thickness of L80 steel with service time under different initial stress: 0 and 0.1 *σ*_s_, respectively.

Equations (20), (30), (34) and (35) were plotted as follows. Figure 12 shows the dependence of the residual wall thickness on service time. Failure points according to the TC and SC are marked on the curves. It can be known that life-span of the L80 downhole tube in Halfaya is 247 months (approximately 20 years) under initial stress of 0.1 *σ*_s_ or 641 months (approximately 53 years) under no initial stress, which indicates that an initial stress of 0.1 yield strength reduces corrosion life by more than half.

## 7. Conclusions

A mathematic model was proposed for predicting life of the downhole tubes. As the premise of this model, corrosion mechanism and corrosion processes of the downhole tubes under different conditions were discussed. Conditions including service environmental parameters and stress as well as their effects on corrosion rate were thought to vary with service duration. Suitability of the model was also discussed. With the model applied, life-span of L80 downhole tubes in Halfaya was predicted. The results show that the life-span is 247 months (approximately 20 years) under initial stress of 0.1 yield strength or 641 months (approximately 53 years) under no initial stress, which indicates that an initial stress of 0.1 yield strength reduces corrosion life by more than half.

## Figures and Tables

**Table 1:** Chemical composition of mineralized water in the medium (g·L^−1^).

NaCl	NaHCO_3_	Na_2_SO_4_	CaCl_2_	MgCl_2_·6H_2_O	pH
236.5	1.01	0.64	26.64	12.68	6

**Table 2:** Corrosion rates of L80 steel under different water contents and different preloading stresses.

Water Content (wt %)	Preloading Stress (*σ*_s_)	No.	Corrosion Rate (mm/a)	Average Corrosion Rate (mm/a)
5	0	1	0.0132	0.0243
		2	0.0354	
	0.5	1	0.0403	0.0311
		2	0.0219	
	0.8	1	0.0663	0.0562
		2	0.0461	
	1.0	1	0.1941	0.2069
		2	0.2197	
30	0	1	0.0329	0.0384
		2	0.0439	
	0.5	1	0.1219	0.1331
		2	0.1443	
	0.8	1	0.3201	0.3015
		2	0.2829	
	1.0	1	0.7584	0.7405
		2	0.7226	
50	0	1	0.0507	0.0431
		2	0.0355	
	0.5	1	0.2484	0.2608
		2	0.2932	
	0.8	1	0.5986	0.5813
		2	0.5640	
	1.0	1	1.3550	1.3322
		2	1.3094	
80	0	1	0.0920	0.0797
		2	0.0674	
	0.5	1	0.3111	0.3845
		2	0.4580	
	0.8	1	0.8316	0.8569
		2	0.8822	
	1.0	1	2.1980	2.2529
		2	2.3078	
100	0	1	0.1002	0.0951
		2	0.0900	
	0.5	1	0.4526	0.4455
		2	0.4384	
	0.8	1	1.0161	0.9870
		2	0.9579	
	1.0	1	2.9331	2.9558
		2	2.9785	
